# Effect of Wearing Surgical Face Masks During Exercise: Does Intensity Matter?

**DOI:** 10.3389/fphys.2021.775750

**Published:** 2021-11-26

**Authors:** Eric Tsz-Chun Poon, Chen Zheng, Stephen Heung-Sang Wong

**Affiliations:** Department of Sports Science and Physical Education, The Chinese University of Hong Kong, Shatin, Hong Kong SAR, China

**Keywords:** masks, exercise test, physical activity, heart rate, lactates, coronavirus disease (COVID)-19

## Abstract

Face masks are widely recommended as means of controlling the coronavirus disease outbreak. This study aimed to examine the physiological and perceptual responses of wearing surgical face masks while exercising at different intensities. Thirteen healthy young adults (mean age, 21.9 ± 1.4 years) conducted randomized crossover trials with or without a surgical face mask. In each trial, participants completed an incremental treadmill protocol, with three 6-min stages (light, moderate, and vigorous at 25, 50, and 75% maximal oxygen uptake, respectively). Physiological outcomes (heart rate, blood lactate, and oxygen saturation level), perceived exertion and discomfort feeling were assessed. No significant differences were observed in physiological outcomes with or without masks at different exercise intensities (*p* > 0.05). However, the rating of perceived exertion (RPE) was significantly higher when exercising vigorously (mask: 15.5 ± 1.5 *vs.* no-mask: 14.2 ± 2.1, *p* < 0.05). Participants wearing masks reported marked discomfort, such as feeling hot, humid, and breathing resistance. Although face mask-wearing during exercise may not have detrimental effects on major physiological parameters, it can increase perceived exertion level and discomfort when the exercise intensity exceeds a certain threshold. Therefore, healthcare professionals should cautiously evaluate each person’s ability to exercise while wearing a mask and tailor their prescription accordingly.

## Introduction

Following the coronavirus disease (COVID-19) outbreak, the use of face masks has become ubiquitous in most countries. The evidence suggests that this strategy can mitigate the current pandemic by reducing the spread of aerosols and respiratory droplets (Leung et al., 2020) and thus decreasing the transmission of the virus (Chu et al., 2020). While the physical and mental health benefits of regular exercise and physical activity (PA) are well documented, recent research has shown that exercise, through its anti-inflammatory effects, may reduce the risk of acute respiratory distress syndrome, a major cause of death in patients with COVID-19 (Yan and Spaulding, 2020). Unfortunately, there has been a decline in global PA as a result of COVID-19 (FitBit, 2020). A recently published longitudinal study by our research group that analyzed PA, before and during COVID-19, reported a significant decline in PA among adults (Zheng et al., 2020).

Virus particles in respiratory droplets may transmit to a greater extent during different forms of PA, including exercise (Blocken et al., 2020; Leung et al., 2020). Although face masks may enable individuals to engage in exercise and PA during the pandemic, there has been a public debate on whether it is safe for individuals to exercise while wearing a face mask, and what should be the recommended exercise intensity for the general population. For instance, it has been speculated that trapping of air in the face mask may compromise oxygen uptake and increase carbon dioxide rebreathing, thereby increasing the arterial carbon dioxide (i.e., hypercapnic hypoxia) that displaces the oxygen from hemoglobin (Chandrasekaran and Fernandes, 2020). The guidelines for wearing face masks during physical activities also vary globally, which may have further confused the fitness and health professionals when prescribing exercise programs. While the World Health Organization (WHO) and Centre for Disease Control and Prevention (CDC) recommend that everyone should wear a face cover when going out in public, both organizations are not in favor of wearing a mask while exercising (CDC, 2021; World Health Organization [WHO], 2021). Specifically, the WHO states that wearing masks during exercise may reduce the individual’s ability to breathe comfortably while the CDC recognizes that it may be difficult to wear a mask during high-intensity PA.

Since the outbreak of COVID-19, several primary studies (Fikenzer et al., 2020; Lassing et al., 2020; Shaw et al., 2020; Doherty et al., 2021; Driver et al., 2021; Epstein et al., 2021) have been conducted and attempted to evaluate the impact of face masks on human physiological and perceptual responses during exercise. While some studies revealed that wearing face mask would pose negative impact on performance and physiological variables (Fikenzer et al., 2020; Lassing et al., 2020; Driver et al., 2021), others demonstrated contrasting results (Shaw et al., 2020; Doherty et al., 2021; Epstein et al., 2021). These disparate findings between studies might be attributed to the notable methodological differences in relevant literature. More importantly, the impact of exercise intensity, a piece of crucial information for optimizing public exercise advice during COVID-19, also appears to be insufficient addressed in previous published work. Given the current lack of evidence-based recommendations for exercising with a face mask, the present study aimed to investigate the physiological and perceptual responses of wearing face masks across different intensities of exercise. It was hypothesized that both physiological and perceptual responses would be negatively affected by wearing a surgical mask, especially during high-intensity exercise.

## Materials and Methods

### Participants

Fourteen healthy, recreationally active young adults (aged 18–25 years) were recruited to participate in the study via advertisements placed at the authors’ university, partner institutions, community centers, and online. The individuals who had severe high blood pressure (≥ 180/100 mmHg) and/or took prescribed medication for chronic disease, myocardial infarction, uncompensated heart failure, or unstable angina pectoris over the previous 6 months (ACSM, 2017), were excluded. All eligible participants were screened by a certified exercise physiologist for a high risk of cardiovascular diseases using a health history questionnaire (ACSM, 2017).

We estimated that a sample size of at least 12 participants would be required to detect an anticipated large effect size (*d* = 0.7) of the perceptual responses between trials, with a power of 0.80 at an alpha level of 0.05 (G*Power version 3.0.10). Detailed explanations of the aim, procedure, benefits and potential risks of the study were provided to the participants and written informed consent was obtained. The study was conducted in accordance with the Declaration of Helsinki, and its protocol was approved by the Ethics Committee of the Chinese University of Hong Kong. All experimental trials were conducted at the Exercise Physiology Laboratory, Department of Sports Science and Physical Education, Chinese University of Hong Kong.

### Preliminary Testing

During the first laboratory visit, participants’ height was measured using a stadiometer (Seca, Leicester, United Kingdom). Bodyweight, body mass index (BMI), and body fat percentage were determined using a body composition analyzer (MC-780MA, Tanita Corp., Tokyo, Japan). The maximal oxygen uptake (VO_2max_) values were assessed during a continuous, incremental, graded uphill treadmill running test to volitional exhaustion, based on a previously reported protocol (Poon et al., 2018). Achievement of VO_2max_ was evaluated based on the following criteria (Edvardsen et al., 2014): (1) a respiratory exchange ratio of≥1.10; (2) failure of heart rate (HR) to increase with an increase in workload; (3) post-exercise blood lactate levels ≥8.0 mmol/L. All participants were able to achieve VO_2max_ based on these criteria. HR was recorded continuously during the test using HR telemetry (H10 Sensor, Polar Electro, Kempele, Finland). The intensity of exercise (as% VO_2max_) for the subsequent experimental trials was based on the corresponding velocity attained during the VO_2max_ test.

### Familiarization Trial

The second visit to the laboratory was a familiarization trial, conducted 1 week after the VO_2max_ test. This trial was intended to familiarize the participants with the experimental procedures and to confirm whether the individually prescribed exercise intensity met the designated percentage VO_2max_ thresholds (25, 50, and 75% VO_2max_) for the experimental trial. Participants were asked to exercise on the treadmill at three predetermined speeds for 6-min each (estimated based on preliminary testing) with oxygen uptake data collected. Adjustments in prescribed speeds were made for the subsequent two experimental trials where appropriate (Sun et al., 2015).

### Experimental Trials

One week after the familiarization trial, the participants completed one of the two experimental trials, with or without a face mask, on a standardized treadmill (Pulsar 3p, h/p/cosmos sports and medical, Germany) in a randomized, crossover order. Participants followed the same incremental treadmill protocol in each trial, with three 6-min stages, for a total of 18 min, at 25% (light), 50% (moderate), and 75% (vigorous) VO_2max_, respectively. No external stimuli (such as music, television, and mobile devices) or verbal encouragement were provided during the trials. The two trials were performed at a 1-week interval. For all trials, the participants arrived at the laboratory at the same time of day (8:00–11:00 a.m.) to eliminate any circadian effects.

The order of the two experimental trials was randomly assigned using an online randomization tool^1^. Typical disposable 3-layer surgical masks with ear loops (IAP Services Ltd., Hong Kong) with an interception function for pollen, bacteria, and dust were used in this study, given that they are the most widely used masks worn by the general population (CHP, 2021). Participants were instructed to follow the manufacturers’ recommendations when putting on the mask, ensuring that it fit snugly over the face (CHP, 2021). Participants were blinded to the test results before completing all trials to avoid the influence of anticipated biases.

### Physiological Measurements

#### Heart Rate

HR telemetry was used for continuous HR monitoring throughout the tests, as described above. Data were recorded at the baseline and across different intensities of exercise (i.e., at the end of each 6-min stage). For analysis purpose, the average of the last 15-s of each stage was used.

#### Blood Lactate Concentration

Blood lactate concentration (BLa) was measured at the baseline and during the last 15 s of each 6-min stage. Capillary blood samples (∼1 μL) were obtained from the fingertips with a portable analyzer (Lactate Plus, Nova Biomedical, Waltham, Massachusetts) (Poon et al., 2020). BLa levels have been commonly used to determine exercise intensities in athletic, normal, and clinical populations (Goodwin et al., 2007; Beneke et al., 2011).

#### Oxygen Saturation

The oxygen saturation level (SpO_2_) was measured non-invasively at the baseline and during the last 15 s of each 6-min stage using a portable finger pulse oximeter (500BL, Zacurate). This marker determined the amount of oxygen bound to hemoglobin in red blood cells within the bloodstream (Jagim et al., 2018).

### Perceptual Measurements

#### Perceived Exertion

Rating of perceived exertion (RPE) was assessed using the Borg scale. The scale ranged from 6 to 20, with anchors ranging from “No exertion at all” (score, 6) to “Maximal exertion” (score, 20). Participants were asked to rate their exertion at the baseline and across different intensities of exercise during the trials (Shariat et al., 2018).

#### Comfort/Discomfort Scale

Participants were asked to indicate their comfort and discomfort using a visual analog scale ranging from 0 to 10, with 0 representing “not at all,” 5 representing “mild” and 10 representing “strong” discomfort. This scale consisted of ten domains, including breathing resistance, tightness, feeling unfit, humidity, heat, odor, fatigue, itchiness, saltiness, and overall discomfort (Li et al., 2005). Five minutes after the mask-wearing trial, participants were asked to indicate how they perceived exercising with a mask during the test.

### Dietary and Exercise Training Control

The participants were requested to avoid strenuous exercise, caffeine, and alcohol for 24 h before all the experimental trials. They were also asked to report their food intake within the previous 24 h at the time of the first trial and then consume the same food on the day before the second trial. All participants were requested to complete a food log and take pictures of the food consumed before each trial. The dietary compliance was confirmed by the investigator.

### Statistical Analysis

Data were analyzed using SPSS (Version 22.0, IBM Corp., Armonk, NY). All continuous variables were presented as means and standard deviations. The mean values of all parameters were assessed for a normal distribution using the Shapiro-Wilk normality test. To determine if parameters differed with masks, the normally distributed parameters (HR, BLa, and SpO_2_) were compared using Repeated measures Analysis of Variance. Non-normally distributed parameters (RPE values) were compared using the Wilcoxon matched-pairs signed ranks test. The *p*-value of 0.05 was considered significant. The effect size was calculated using Cohen’s d to indicate the magnitude of the difference between two means, where appropriate (Cohen, 1992). Scores of 0.2, 0.5, and > 0.8, were considered small, moderate, and large effect sizes, respectively.

## Results

Thirteen participants (male, 7; female, 6) completed all required testing and their data were included in the subsequent analysis. One participants withdrew from the study unexpectedly owing to an injury unrelated to the study. The mean age of the participants was 21.9 ± 1.4 years. Other demographic characteristics, including height, weight, body fat percentage, VO_2max_, and HR_max_, are presented in Table 1. No differences in baseline levels were observed in any of the parameters during the two trials.

**TABLE 1:** Demographic characteristics of the participants.

	Male (*n* = 7)	Female (*n* = 6)	Total (*n* = 13)
Age (year)	22.11.6	21.71.2	21.91.4
Height (cm)	174.26.3	161.98.2	168.59.4
Weight (kg)	68.96.4	52.34.4	61.210.1
BMI (kg/m^2^)	22.72.1	20.01.0	21.62.1
Body fat (%)	12.42.8	24.73.1	18.17.0
VO_2m*ax*_ (mL/kg/min)	52.13.3	36.83.6	45.08.6
HR_*max*_ (bpm)	186.19.1	186.87.4	186.58.0

*BMI, body mass index; bpm, beats per minute; HR_max_, maximal heart rate; VO_2max_, maximal oxygen uptake.*

### Physiological Measurements

**FIGURE 1:**
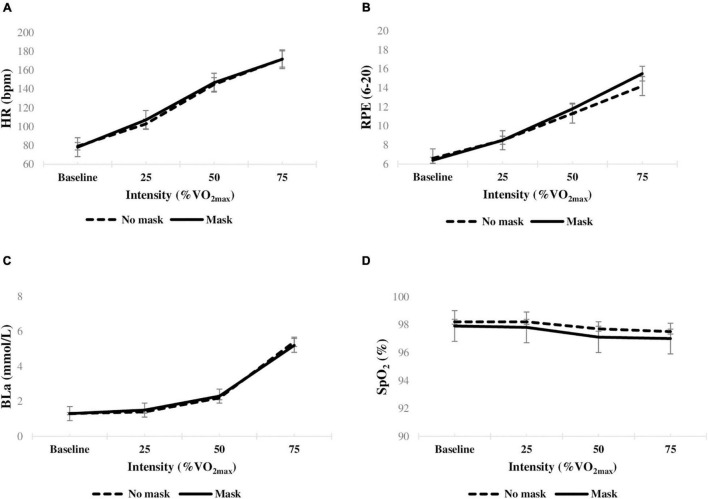
Mean changes in physiological and perceptual parameters (*n* = 13) during exercise with and without a surgical mask. **(A)** HR heart rate; **(B)** RPE rating of perceived exertion; **(C)** BLa blood lactate concentration; **(D)** SpO_2_ oxygen saturation level. *p < 0.05.

A similar increase in the HR was observed during the exercise session between the masked and unmasked conditions, reaching approximately 92% HR_max_ at the end of both trials. Increment in BLa was also similar across different intensities of exercise during both trials. The SpO_2_ levels remained within the normal physiological range (95–100%) throughout the session, indicating no sign of hypoxia. No significant differences in physiological outcomes were evident between trials at any exercise intensity (*p* > 0.05, Table 2 and Figure 1).

**TABLE 2:** Mean physiological responses and perceived exertion.

Measures	Intensity	No mask	Mask	Difference (95% CI)	Cohen’s d effect size	*p*-value
HR (bpm)	Baseline	79.214.3	78.2 ± 12.6	1.0 (−5.2 to 7.2)	0.07	0.73
	LIG	102.811.2	107.1 ± 11.8	−4.2 (−8.5 to 0.3)	0.34	0.06
	MOD	144.814.1	146.7 ± 11.7	−1.8 (−5.0 to 1.4)	0.15	0.24
	VIG	171.89.4	171.6 ± 9.2	0.15 (−1.5 to 1.8)	0.02	0.84
RPE (6–20)	Baseline	6.60.9	6.4 ± 0.7	0.2 (−0.2 to 0.7)	0.25	0.26
	LIG	8.51.3	8.5 ± 1.6	0.0 (−0.7 to 0.7)	0.00	0.93
	MOD	11.31.9	11.8 ± 2.1	−0.5 (−1.5 to 0.4)	0.25	0.18
	VIG	14.22.1	15.5 ± 1.5*	−1.3 (−2.2 to −0.4)	0.71	< 0.001*
BLa (mmol/L)	Baseline	1.30.4	1.3 ± 0.3	0.0 (−0.3 to 0.4)	0.00	0.84
	LIG	1.40.7	1.5 ± 0.5	−0.1 (−0.5 to 0.3)	0.16	0.52
	MOD	2.20.8	2.3 ± 0.8	−0.1 (−0.5 to 0.4)	0.13	0.68
	VIG	5.41.7	5.2 ± 1.6	0.2 (−1.0 to 1.4)	0.12	0.69
SpO_2_ (%)	Baseline	98.20.9	97.9 ± 1.1	0.3 (−0.2 to 0.8)	0.30	0.22
	LIG	98.20.8	97.8 ± 1.1	0.4 (−0.5 to 1.3)	0.41	0.36
	MOD	97.71.7	97.1 ± 1.3	0.6 (−0.4 to 1.6)	0.40	0.19
	VIG	97.50.8	97.0 ± 1.4	0.5 (−0.3 to 1.2)	0.44	0.19

*LIG: light intensity (25%VO_2max_); MOD: moderate intensity (50%VO_2max_); VIG: vigorous intensity (75%VO_2max_). BLa, blood lactate concentration; RPE, rating of perceived exertion; SpO_2_, oxygen saturation level.*

*p < 0.05. Significant difference compared to no mask.

### Perceptual Measurements

For the RPE data, no significant differences were observed between the low and moderate intensities (*p* > 0.05), but the rating was significantly higher when exercising at a vigorous intensity (mask: 15.5 ± 1.5 *vs*. no mask: 14.2 ± 2.1, *p* < 0.05, Table 2 and Figure 1). Cohen’s d statistics indicated a moderate effect size (*d* > 0.7) between the groups. Participants reported marked discomfort when wearing a mask, especially for the hot (6.8 ± 2.0), humid (7.2 ± 1.9), and breathing resistance (7.5 ± 1.7) (Table 3).

**TABLE 3:** Perceived discomfort level when wearing the face mask during the test.

Domain	0 (no discomfort at all) to 10 (maximal discomfort)
Humid	7.2 ± 1.9
Hot	6.8 ± 2.0
Breath resistance	7.5 ± 1.7
Itchy	3.7 ± 3.2
Tightness	4.4 ± 2.6
Salty	2.4 ± 2.5
Unfit	2.8 ± 2.3
Odor	2.5 ± 2.1
Fatigue	6.1 ± 1.6
Overall	5.8 ± 2.1

## Discussion

The major finding of the present study was that wearing a surgical face mask during exercise does not appear to have a detrimental effect on all measured physiological outcomes. However, an increased level of perceived exertion was reported on mask-wearing, as the intensity reached a vigorous level (≥75% VO_2max_). This study expanded the current body of evidence on face mask and exercise by examining the effect of wearing surgical masks, the most commonly worn mask by the general population, across different exercise intensities.

Our findings were contrary to our hypothesis that physiological parameters would be negatively affected by wearing a surgical face mask. This hypothesis was based on an earlier commentary (Chandrasekaran and Fernandes, 2020), that exercising with face masks may pose significant health risks and burden on various physiological systems, such as the pulmonary, circulatory, and immune systems, due to hypercapnia (i.e., increase in arterial carbon dioxide). Evidence from previous studies supports these physiological effects. For example, a recent study that examined the impact of wearing a surgical face mask vs. N95 face mask on cardiopulmonary exercise capacity in 12 healthy men during an incremental maximal exercise test found a significant reduction in their pulmonary function and ventilation with both masks; they also observed reduction in the cardiopulmonary exercise capacity (Fikenzer et al., 2020). Lassing et al. (2020) tested the effects of a surgical face mask on cardiopulmonary parameters during exercise at maximal lactate steady state in 14 healthy men. The use of surgical face masks led to an increase in airway resistance and HR during exercise sessions. Similarly, another study (Driver et al., 2021) observed a significant decline in VO_2max_, minute ventilation, and HR_max_ during a graded treadmill running test in 31 healthy adults wearing a cloth mask.

However, results from the above three studies should be taken with caution, as in all these experiments, a spirometry mask, to assess gas exchange data, was worn over the face mask, which may have sealed the face mask and affected the external validity of the measurements. It is possible that the extra pressure exerted by the spirometry apparatus over the face mask significantly increased the overall resistance to airflow from the mouth and trapping of air, exacerbating the extent of carbon dioxide rebreathing and associated physiological consequences. Furthermore, it is also likely that wearing the face mask and spirometry mask simultaneously would affect the measurement of ventilation and potentially expired gases. For instance, previous work by Fikenzer et al. (2020) showed minimal reduction in the total work achieved and no change in peak cardiac output, blood lactate levels, or bloods gases, yet a substantial (∼30%) reduction in ventilation and VO_2max_. The data did not seem to be internally consistent, as one would expect a larger decrement in power output, and changes in associated physiological outcomes with reduced alveolar ventilation arising because of increased work of breathing. Future studies should thus be aware of the potential flaws or biases in data collection when a face mask is worn together with a spirometry apparatus for gas collection purposes, in which greater restriction to breathing and interference to expired gas measurement might have imposed.

In contrast, other experiments, that did not require the use of spirometry masks, tended to show less or even no detrimental effect on most physiological outcomes, which is consistent with our study findings. When expressed relative to peak exercise performance, Shaw et al. (2020) showed that no differences were evident between masked or unmasked conditions for arterial oxygen saturation, tissue oxygenation index, or HR at any time during a cycle ergometry test to exhaustion. In another study by Epstein et al. (2021), 16 healthy men performed a maximal exercise test without a mask, with a surgical mask, and with an N95 respirator, using a standard cycle ergometry ramp protocol. They found that HR, respiratory rate, blood pressure, and time to exhaustion did not differ between groups. Doherty et al. (2021) examined the impact of wearing cloth or surgical masks on the cardiopulmonary responses in 12 healthy young individuals during an 8-min moderate-intensity cycling exercise. It was found that wearing surgical or cloth masks during exercise had no impact on breathing frequency, tidal volume, oxygenation, and heart rate. Taken together, these findings suggest that in healthy individuals, the physiological impact of wearing masks on short-term light–vigorous-intensity exercise appears to be modest.

While no evident physiological difference was observed in the present study, perceived exertion was significantly higher when exercising at vigorous intensity. This observation was in agreement with that of a previous study (Driver et al., 2021), where participants wearing a cloth face mask reported shortness of breath and claustrophobia at higher exercise intensities. In our study as well, participants, when wearing a mask, reported marked discomfort, including feeling hot, humid, and resistance on breathing. Due to such uncomfortable feelings, the individuals may find it harder to exert maximum effort and fatigue more quickly with increasing exercise intensity (Driver et al., 2021). Our study raises particular concern for individuals exercising in a hot and humid environment. Once surgical masks become wet during exercise, they may break down and subsequently lose the ability to block outgoing viruses and other germs (World Health Organization [WHO], 2021). The retained moisture from the exhaled breath and facial sweat accumulation within the mask can also result in a loosening of its seal to the face and a potential increase in breathing resistance due to blockage of pores in the mask that could increase the work of breathing (Roberge et al., 2012). As a result, the subjective discomfort associated with mask-wearing, increased facial temperature, and increased difficulty in breathing (i.e., sense of dyspnea) at higher intensities of physical activity may lead to a perceptual cue for early termination of exercise (Driver et al., 2021).

This study had several strengths— the effects of surgical face masks evaluated using a randomized research design and focus on a range of exercise intensities by standardizing individuals’%VO_2max_. Using accurate and reliable measures of physiological and perceptual parameters and controlling participants’ diet and physical activity also strengthened our data quality. In addition, the test protocol employed in the current study had a higher external validity when compared with previous studies in which spirometer masks were placed over the test masks. Thus, our results provide genuine impact and real-life implications of wearing face masks when exercising. Despite these strengths, there are certain limitations of the present study. We only recruited young, apparently healthy individuals, hence, the results should be generalized to other populations groups (such as children, older adults, sedentary, or with clinical conditions) with caution. Further, we acknowledge that significant variability (such as in material, shape, and design) exists between face masks used by the public, and each of these factors may have independent effects on exercise-related responses. For instance, recent evidence suggested that wearing a cloth face mask (i.e., also common for people to use for exercising) may increase dyspnea more than wearing a surgical face mask during vigorous exercise, potentially due to differences in breathing resistance and tightness exerted by these two types of masks. These factors should been addressed thoroughly in future studies (Fukushi et al., 2021). However, we do believe that our current findings provide valuable information that will assist in formulating evidence-based exercise recommendations related to mask-wearing in the current pandemic.

To conclude, this study provides a more comprehensive understanding of the physiological and perceptual effects of exercising with a mask across different intensities. Wearing a surgical face mask during exercise does not appear to have detrimental effects on heart rate, blood lactate level, and oxygen saturation level, but may increase perceived exertion level and discomfort when the exercise intensity exceeds a certain threshold in healthy individuals. From a practical point of view, these data provide valuable information for formulating appropriate health care policies and optimizing exercise recommendations for the public during COVID-19. Healthcare professionals should therefore cautiously evaluate each person’s ability to exercise while wearing a mask and consider adjusting the prescription (including frequency, intensity, time, and type of exercises) accordingly. Further research in wider populations is warranted to determine how wearing various types of face masks while exercising may impact human body responses in different environments.

## Data Availability Statement

The raw data supporting the conclusions of this article will be made available by the authors, without undue reservation.

## Ethics Statement

The studies involving human participants were reviewed and approved by the Ethics Committee of the Chinese University of Hong Kong. The patients/participants provided their written informed consent to participate in this study.

## Author Contributions

EP, CZ, and SW participated in the conception, responsible for the writing and finalization of the manuscript, and design of the study. EP was responsible for testing. EP and CZ were responsible for data collection and statistical analysis. All authors contributed to the manuscript and approved the submitted version.

## Conflict of Interest

The authors declare that the research was conducted in the absence of any commercial or financial relationships that could be construed as a potential conflict of interest.

## Publisher’s Note

All claims expressed in this article are solely those of the authors and do not necessarily represent those of their affiliated organizations, or those of the publisher, the editors and the reviewers. Any product that may be evaluated in this article, or claim that may be made by its manufacturer, is not guaranteed or endorsed by the publisher.

## Footnotes

1
https://www.random.org/lists/
